# Proteo-Transcriptomic Dynamics of Cellular Response to HIV-1 Infection

**DOI:** 10.1038/s41598-018-36135-3

**Published:** 2019-01-18

**Authors:** Monica Golumbeanu, Sébastien Desfarges, Céline Hernandez, Manfredo Quadroni, Sylvie Rato, Pejman Mohammadi, Amalio Telenti, Niko Beerenwinkel, Angela Ciuffi

**Affiliations:** 1 Department of Biosystems Science and Engineering, ETH Zurich, Basel, Switzerland; 2 SIB Swiss Institute of Bioinformatics, Basel, Switzerland; 3 Institute of Microbiology, Lausanne University Hospital and University of Lausanne, Lausanne, Switzerland; 4 InvivoGen, Toulouse, France; 5 Center for Integrative Genomics, University of Lausanne, Lausanne, Switzerland; 6 Computational Systems Biology Team, Institut de Biologie de I’Ecole Normale Supérieure, CNRS UMR8197, INSERM U1024, ENS, PSL Université, Paris France; 7 Department of Integrative Structural and Computational Biology, The Scripps Research Institute, La Jolla, (CA) USA

## Abstract

Throughout the HIV-1 replication cycle, complex host-pathogen interactions take place in the infected cell, leading to the production of new virions. The virus modulates the host cellular machinery in order to support its life cycle, while counteracting intracellular defense mechanisms. We investigated the dynamic host response to HIV-1 infection by systematically measuring transcriptomic, proteomic, and phosphoproteomic expression changes in infected and uninfected SupT1 CD4+ T cells at five time points of the viral replication process. By means of a Gaussian mixed-effects model implemented in the new R/Bioconductor package TMixClust, we clustered host genes based on their temporal expression patterns. We identified a proteo-transcriptomic gene expression signature of 388 host genes specific for HIV-1 replication. Comprehensive functional analyses of these genes confirmed the previously described roles of some of the genes and revealed novel key virus-host interactions affecting multiple molecular processes within the host cell, including signal transduction, metabolism, cell cycle, and immune system. The results of our analysis are accessible through a freely available, dedicated and user-friendly R/Shiny application, called PEACHi2.0. This resource constitutes a catalogue of dynamic host responses to HIV-1 infection that provides a basis for a more comprehensive understanding of virus-host interactions.

## Introduction

Upon cellular invasion of the host T-cell, the success of HIV-1 infection depends on numerous virus-host interactions. During the roughly 24-hour-long replication cycle, HIV-1 enters the host cell, integrates its genome, and utilizes the host cellular machinery in order to produce new virions. The host genes that are utilized by the virus to support its lifecycle are called HIV dependency factors (HDFs)^1–9^. Conversely, the host cell defense system tries to counteract infection through innate immune cellular responses, attempting to block different stages of the replication cycle. The host genes involved in these defense responses are called HIV inhibitory factors (HIFs) and include virus-specific restriction factors^10–13^.

**Figure 1:**
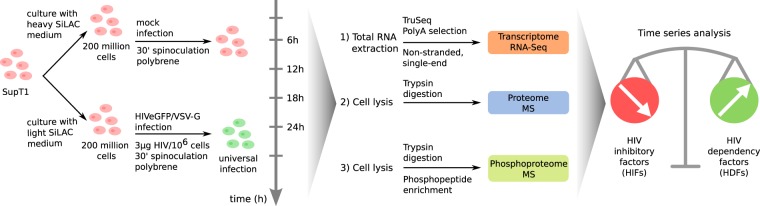
Experimental design overview. SupT1 CD4+ T cells were cultured in a heavy (^13^C_6_-L-lysine, ^13^C_6_^15^N_4_-L-arginine) or light isotope culture medium, respectively. Cells were either Mock-infected, or infected with an HIV-GFP-based vector. At different time points, cells were collected for viral intermediate measurements and for omic (transcriptome, proteome and phosphoproteome) measurements. The dynamic interplay of host-HIV interactions was investigated at the three different molecular levels, aiming at identifying novel putative host factors modulated during HIV replication. MS: mass spectrometry.

Several studies have investigated virus-host interactions and identified dependency and inhibitory factors, based on siRNA experiments^1–5,8,9,14^, proteomic assays^6,7,15^, and functional screens^16,17^. Most of these analyses were focused on one type of omics data, either transcriptomics or proteomics, at a single time point. Only a few studies investigated HIV-host interactions with a temporal resolution^18–20^. In order to gain a more comprehensive understanding of virus-host interactions over time, we explored virus-induced cellular reprogramming at multiple molecular levels and time points. A series of high-resolution, genome-wide measurements of the transcriptome, proteome, and phosphoproteome were conducted in uninfected and infected SupT1 CD4+ T cells in order to define the dynamic, integrated proteo-transcriptomic response of the cell to infection with an HIVeGFP vector and to understand the key molecular players maintaining a balance between host support for viral replication and host defense response to inhibit infection (Fig. 1). For this purpose, we built on previous work and used a well-established SupT1 experimental cellular system^18^.

Using a clustering approach that we implemented in the R/Bioconductor package TMixClust, we identified expression signatures specific to HIV-1 infection, corresponding to novel putative host factors involved in HIV replication. Functional analysis of these genes provided insight into how the virus modulates key host molecular processes to assure completion of its replication cycle. Our results constitute a unique resource, presenting for the first time a temporal analysis of HIV infection jointly at three different omics levels, namely, transcriptome, proteome and phosphoproteome. The data and results are freely accessible through an online catalogue allowing users to inspect and use them for future research, available at https://peachi2.shinyapps.io/peachi2/.

## Results

### Analysis of HIV replication dynamics

**Figure 2:**
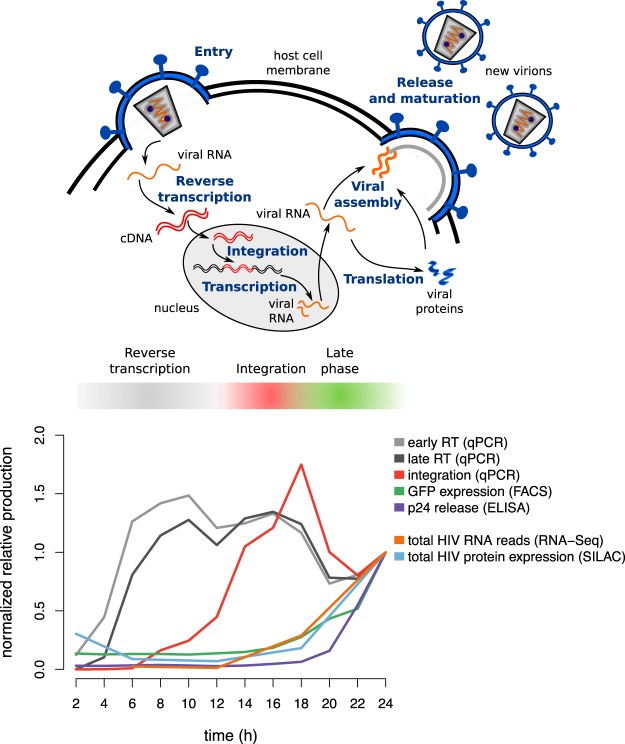
HIV replication cycle progression. (Upper panel) Schematic representation of the HIV-1 replication cycle illustrating the major steps of HIV progression. (Lower panel) Relative quantification of viral intermediates, transcription and protein synthesis normalized by the 24 h time point and depicting the three key stages of HIV life cycle: reverse transcription, integration and late phase. Viral intermediate measurements consist of multiple viral DNA forms, *i*.*e*. early and late reverse transcription products (early RT, late RT) as well as integrated viral DNA measured by qPCR, expression of the virally encoded GFP protein measured by FACS (geometric mean fluorescence intensity), and viral particle production measured by p24 ELISA of the supernatant. Total viral transcription was quantified by the RNA-Seq reads which were aligned to the HIV genome. Total viral protein expression was quantified by mass spectrometry.

SupT1 CD4+ T cells were either Mock-infected or infected with an HIVeGFP-based vector. Virus-host interactions were characterized over time by multiple time series measurements (Fig. 1). As described previously^18^, the infection conditions allowed for quasi universal infection. Through an initial quantitative analysis, levels of viral intermediates - early and late reverse transcription (RT) products, integrated viral DNA, viral translation (GFP expression), and viral particle release (p24 release) - were evaluated every 2 h during 24 h to ascertain successful infection and follow HIV replication cycle advancement (Fig. 2). Consistent with our previous analysis^18^, we observed a prevalence of early and late reverse transcription products corresponding to the reverse transcription stage peaking at ~10 h post infection. Reverse transcription was followed by the integration stage indicated by a peak of integrated viral DNA at ~18 h. Finally, emergence of viral translation and viral particle release during 18–24 h post infection signaled the final stage of the viral replication cycle (Fig. 2). Analysis of joint virus-host omics data was performed every 6 h at the transcriptome level via RNA-Seq, and at the proteome and phosphoproteome levels via mass spectrometry (MS), quantifying total viral RNA, protein production, and phosphorylation events over time (Supplementary Figs S1 and S2). The viral transcript and protein production measurements over time from the virus-host omics data were consistent with the quantitative analysis of HIV replication progression through viral intermediate measurements (Fig. 2). Precisely, a considerable increase in viral transcripts and protein products after 18 h was observed, delineating the final stage of the viral replication cycle.

### Quantification of omics measurements in HIV- and Mock-infected SupT1 cells

**Figure 3:**
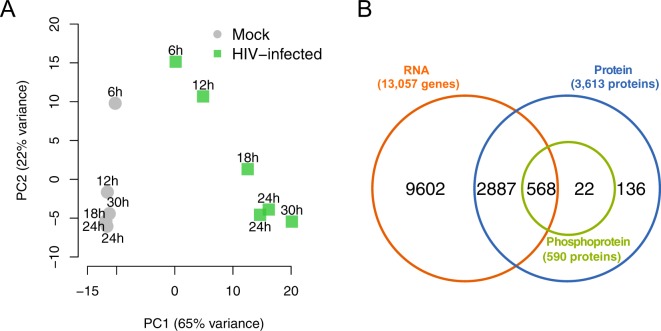
Overview of the time series omics quantification of Mock and HIV-infected SupT1 cells. (**A**) Principal component analysis for HIV and Mock gene expression profiles obtained from RNA-Seq. Each data point corresponds to the ensemble of gene expression RNA-Seq read counts for a corresponding time point and condition (either Mock or HIV-infected). The time-points are indicated next to each data point, specifying whether the corresponding sample is a Mock (grey circles) or HIV-infected (green squares) sample. Non-infected samples (Mock) cluster together and separate from the HIV-infected samples which are consistently distributed according to their time after HIV-infection. (**B**) Venn diagram representing the numbers of detected, filtered genes at RNA, protein, and phosphoprotein levels, as well as the overlap of detected genes between these levels.

RNA-Seq yielded on average 108 million raw sequencing reads per sample. After read trimming and quality filtering, we obtained an average of 102 million aligned reads per sample, including uniquely aligned reads and multimapper reads. In each sample, over 90% of the reads were uniquely mapped to the concatenated human plus HIV reference sequence (Supplementary Table S1, Supplementary Fig. S3A). The number of reads mapping to the HIV genome increased with time, and was significantly correlated with viral translation (Pearson correlation *ρ* = 0.97, Pearson correlation test: *p* = 0.03) and with viral particle release (Pearson correlation *ρ* = 0.99, Pearson correlation test: *p* = 0.007), consistent with HIV life cycle progression (Supplementary Fig. S3B). Following transcriptome profiling and filtering, HIV versus Mock RNA expression changes at five time points were obtained for 13,057 genes (Supplementary File S1, Supplementary Table S2). Principal component analysis of HIV and Mock RNA-Seq gene expression read counts resulted in a clear separation between HIV and Mock samples, as well as a temporal ordering of the HIV-infected samples (Fig. 3A).

Following SILAC MS data analysis, we detected 4,947 proteins and 2,072 phosphorylation sites corresponding to 793 proteins (Supplementary Table S2). After processing and filtering, HIV/Mock time series protein expression profiles were obtained for 3,613 proteins and 1,111 phosphorylation sites corresponding to 590 proteins (Supplementary Table S2, Supplementary File S1). We observed a similar temporal ordering of the protein and phosphoprotein samples in a principal component analysis of the log_2_(HIV/Mock) protein and phosphoprotein ratios (Supplementary Fig. S4).

In total, 3,455 genes had measurements at both RNA (out of 13,057 transcripts, corresponding to 26.5%) and protein (out of 3,613 proteins, corresponding to 95.6%); 568 genes had measurements at all three levels (Fig. 3B).

### Proteo-transcriptomic temporal analysis of the host cell upon HIV-1 infection

Differential expression analysis between Mock and HIVeGFP-infected conditions was performed initially regardless of the time point (cf. Methods) and resulted in 1506 differentially expressed genes, 415 differentially expressed proteins, and 157 differentially expressed phosphorylation sites, corresponding to 125 proteins with a differential phosphorylation status (Supplementary Fig. S5, Supplementary File S2). Using a Gaussian mixed-effects models framework, we modeled the changes in expression of the differentially expressed genes through time and clustered the genes at each omics level based on their temporal profiles (cf. Methods). We implemented the model in the dedicated R/Bioconductor package TMixClust. For each data type (RNA, protein, and phosphoprotein), stability and silhouette analyses indicated that the number of Gaussian model components, or clusters, that provided the optimal segregation with the largest average silhouette width was two (Supplementary Figs S6–S9). Further investigation of the time series behavior in each cluster revealed a global upregulation and downregulation expression pattern for each data type (Supplementary Fig. S10). We found 1073 upregulated and 433 downregulated genes at the RNA level, 206 upregulated and 209 downregulated proteins, and 24 upregulated and 133 downregulated phosphorylation sites corresponding to 20 and 100 unique genes, respectively (Supplementary File S3).

The results of the clustering analysis define the proteo-transcriptomic expression response patterns of host genes to HIV-1 infection. These patterns and their underlying data are available in an R/Shiny application providing a user-friendly querying platform, coined PEACHi2 (Patterns of Expression and Analysis of Clusters of HIV/Host interactions v2.0), accessible at https://peachi2.shinyapps.io/peachi2/. On this platform, users can specify the desired proteo-transcriptomic behavior (up- or downregulation patterns at the three data levels) and retrieve the corresponding genes, as well as inspect the behavior of custom gene lists and download the associated data (Supplementary Fig. S11).

**Figure 4:**
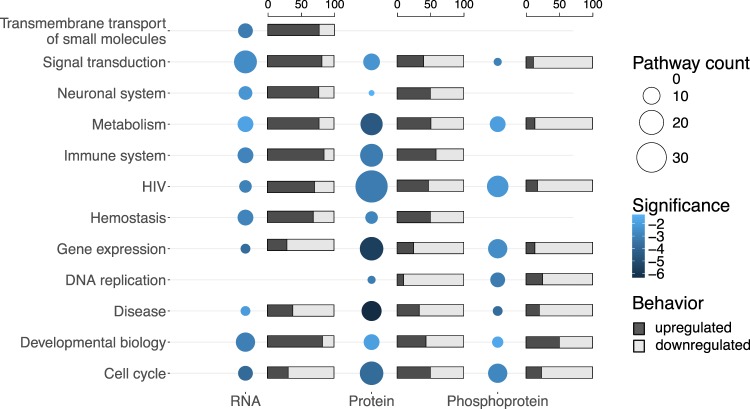
Enrichment analysis of differentially expressed genes. Differentially expressed genes identified at RNA, protein and phosphoprotein levels were analyzed in terms of enrichment in Reactome pathways, as well as in HIV-related pathways included in a separate collection. Enriched Reactome pathways were grouped according to the Reactome hierarchy into enriched categories, while the HIV-related pathways were assigned to a separate category called “HIV”. Category names are specified on the left side of the figure. Each circle size (Pathway count) is proportional to the number of enriched pathways included in the corresponding category. The color of each circle (Significance) reflects the log10 of the geometric mean of the corrected *p*-values for the pathways included in each category. Next to each circle, the barplot displays the percentages of upregulated and downregulated genes in the corresponding category.

We functionally characterized the differentially expressed genes in terms of gene set enrichment in Reactome pathways^21^, as well as in a curated library of gene sets associated to HIV-related processes, available at^18^ (Supplementary File S4). The enriched Reactome pathways were stratified into functional categories according to the first layer of the Reactome hierarchy of pathways^21^. A separate category, named “HIV”, represented the above-mentioned library of HIV-related gene sets. The differentially expressed genes involved in these functional categories were further assessed according to their identified infection-specific patterns of up- and downregulation, leading to a comprehensive image of how host gene expression is modulated during HIV infection at multiple molecular levels (Fig. 4). We observed a pronounced upregulation response at the RNA level, for the most part involving signal transduction pathways, as opposed to the other two omics levels. Our data suggest that HIV-1 influences the signaling mechanisms of infected cells mainly by affecting expression of cell surface receptors, chemokine receptors, G-protein-coupled receptors, or genes involved in signaling of Rho GTPases (Supplementary File S5), recapitulating previous findings^22–25^. At the protein and phosphoprotein levels, the identified differentially expressed genes were mainly enriched in pathways describing immune system, metabolism, gene expression, cell cycle, and HIV-related processes (Fig. 4, Supplementary File S5).

Four genes were found differentially expressed at all three omics levels, specifically the histone gene HIST1H1B, cytoskeleton protein LIMA1, transcription factor TFAP4, and the uracil DNA glycosylase UNG (Supplementary Fig. S12). Although the exact roles of these genes throughout the HIV replication cycle are insufficiently understood, their interactions with viral genes have been reported by previous studies. With a putative role in adjusting nucleosome structure and chromatin organization during HIV-1 infection, HIST1H1B has been shown to interact with HIV proteins Gag and Tat^26,27^. Co-immunoprecipitation assays have identified interactions between LIMA1 and viral protein Tat^28^. Downregulation of TFAP4 at protein level observed in our data has also been previously reported^19^. Finally, it has been shown that HIV gene Vpr induces UNG degradation during HIV infection^29,30^, supporting its observed downregulation in our data.

**Figure 5:**
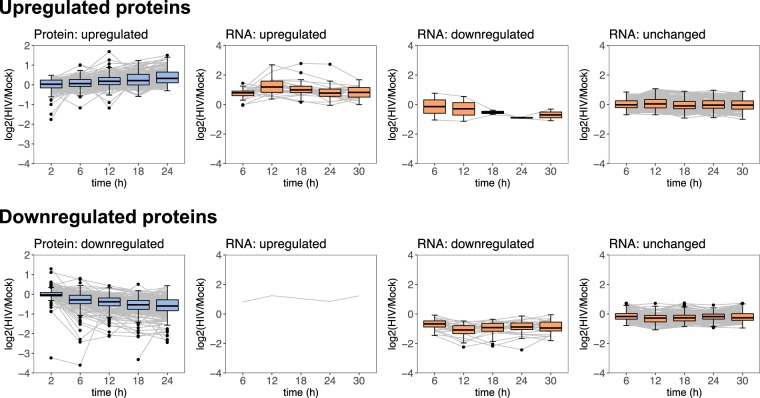
Time series patterns specific to putative host factors regulated during HIV replication. Two distinct patterns of protein expression behavior may define candidate host factors involved in HIV replication: 192 upregulated proteins (upper panel) and 196 downregulated proteins (lower panel). Gene expression dynamics of these factors differs between RNA and protein levels. Each line corresponds to a time series expression of a single gene at RNA and protein levels. The box plots display the gene expression distributions at each time point for RNA (orange) and protein (blue).

Based on their protein up- or downregulation behavior, host genes were nominated as putative host factors which are modulated during HIV replication (Supplementary File S6). Table 1 and Fig. 5 summarize the observed proteo-transcriptomic patterns associated to these factors.

**Table 1:** Proteo-transcriptomic patterns putatively associated to host factors involved in HIV replication.

Category	Proteo-transcriptomic pattern		Number of genes
	RNA	Protein	
Upregulated host proteins	Upregulated	Upregulated	23
	Downregulated		2
	No change		167
	**Total**: **192 genes**		
Downregulated host proteins	Upregulated	Downregulated	1
	Downregulated		25
	No change		170
	**Total**: **196 genes**		

The table presents a set of defined proteo-transcriptomic dynamic patterns presumably corresponding to host genes that play a role in HIV replication. Each row is associated to a specific pattern and contains the number of genes displaying the respective behavior at RNA and protein level. The phosphoproteome measurements were not used for defining these patterns, as the corresponding data was presenting a high amount of missing values and few genes with differentially expressed phosphorylation (125 differentially phosphorylated genes, with only 22 also differentially expressed at RNA or protein level). At the end of each category, the total number of genes is specified.

We first assessed to what extent our detected putative factors reproduce previous well-known HIV-host interactions. For this reason, a list of 3,743 previously reported host genes presumably involved in HIV-1 replication was established by including reported candidates from published studies. In this list, we included genes reported in siRNA screens^1–3,5,8,16^, protein assays^6,7,15^, or genome-wide analyses^9,31^ (Supplementary Table S3, Supplementary File S7). We observed a statistically significant overlap between this list and our selected candidate factors. More precisely, 57 upregulated (29.7%) and 73 downregulated factors (37.2%), corresponding to a total of 130 out of 388 (33.5%) candidate factors were previously reported as being involved in host cell gene expression modulation during HIV replication (Hypergeometric test: *p* < 10^−47^, Supplementary File S7). Among these, we recovered well-known, hallmark cellular genes involved in virus-host interactions, such as the surface receptor CD4^32^, the transcription regulator NFKB1^33^, the cyclin-dependent kinase CDK9^34^, the Vpr-binding protein VPRBP^35^, and the uracil DNA glycosylase UNG^36^. In addition to the previously reported factors, our time series proteo-transcriptomic analysis identified new genes, including DEAD-box helicases, zinc-finger proteins, and kinesins, potentially involved in HIV replication, that have not been described in this role before (Supplementary File S6). The PEACHi2 online resource allows retrieving all the identified candidate factors and studying their behavior at the three omics levels.

Enrichment analysis of the selected upregulated and downregulated host proteins revealed the functional layers of the host cellular machinery that are perturbed during HIV infection. Precisely, these proteins were found enriched mainly in pathways associated to HIV, metabolism, cell cycle, immune system, and gene expression (Supplementary Fig. S13, Supplementary File S8). Moreover, approximately 14% of the downregulated proteins consisted of nucleolar and ribosomal proteins involved in ribosome biogenesis. Their observed downregulation behavior has also been reported in previous studies and associated to HIV mechanisms of controlling host protein synthesis, ultimately inducing apoptosis at late stages of viral replication^37^. Enriched immune responses differed between the two categories of factors. While upregulated proteins were enriched in pathways mainly consisting of major histocompatibility complex (MHC) class I and II presentation, TOLL-receptor cascades and cytokine signaling, downregulated proteins were enriched in pathways containing four innate immune genes (CNPY3, PPP2R5D, CASP2, and CD4) as well as genes involved in CD28 family co-stimulation, CTLA-4 inhibitory signaling, TCR signaling, and translocation of ZAP70 to the immunological synapse (Supplementary File S8). Regulatory changes within these pathways during HIV infection have been reported in previous studies as well^38–41^. Furthermore, the immune system gene CD4 was among the reported downregulated factors, in agreement with its well-known interaction with the viral protein Nef that downregulates CD4^32^.

The majority of genes in both factor classes exhibited differential expression behavior at the protein level but not at the RNA level, suggesting direct modulation of these host proteins by HIV (Fig. 5, Supplementary File S6). Such examples include the transcription factors TCF7 and LEF1 which were not found differentially expressed at the RNA level, but downregulated at the protein level. This behavior is consistent with direct inhibition at the protein level of the Wnt/β-catenin pathway during HIV replication, as reported previously^42,43^. Conversely, only 13% upregulated and 13.3% downregulated factors were differentially expressed at both levels (Table 1). Among these were four immune system genes with consistent behavior at RNA and protein levels: upregulated LCP2, and downregulated GNA15, KIF20A, and TRIM36. LCP2 (Lymphocyte Cytosolic Protein 2) is involved in TCR-mediated intracellular activation and was shown to favor HIV replication following siRNA functional studies^1,44^. Furthermore, it has been shown that the HIV envelope protein upregulates LCP2^45^. These previously reported results are concordant with the observed upregulation behavior in our data, at RNA and protein levels. GNA15, a G protein subunit, and KIF20A, a kinesin protein, were not reported so far to be involved in the HIV replication cycle. In contrast, TRIM36, a tripartite family member, was shown to be downregulated by the viral protein Vif. However, the exact role of TRIM36 remains to be elucidated.

### Time series host phosphoproteomic characterization during HIV-1 infection

We found 157 differentially expressed phosphorylation sites between HIV- and Mock-infected conditions, corresponding to 125 unique phosphorylated proteins (Supplementary Table S2) involved in host molecular processes such as signal transduction, cell cycle, metabolism, and gene expression (Fig. 4, Supplementary File S5). Out of these, 24 sites presented increased phosphorylation (21 unique proteins) and 133 sites were down-phosphorylated (113 unique proteins). Only 22 of the differentially phosphorylated proteins were also found differentially expressed at RNA and protein levels (Supplementary File S9).

Few studies have analyzed the host phosphoproteomic modulation during infection with HIV^15,19^ and little is known about the regulation of the host gene phosphorylation by HIV. Wojcechowskyj *et al*. have performed a phosphoproteomic screen during HIV-1 entry and have identified 175 differentially phosphorylated proteins and validated 37 of them through siRNA-induced depletion^15^. Their study brings forward and demonstrates the importance of serine/arginine-rich proteins for HIV-1 splicing. Our time series phosphoproteomic data recovers 21 out of the 175 differentially phosphorylated proteins found by their analysis, with 7 of them being among the ones validated by their siRNA experiments (Supplementary File S9). These include serine/arginine-rich proteins ACIN1 (Apoptotic Chromatin Condensation Inducer 1) and PNN (Pinin).

In another study of phosphoproteome regulation during HIV infection, Greenwood *et al*. show that the viral protein Vif induces degradation of PPP2A phosphatase family members by phosphorylation^19^. Among the differentially phosphorylated proteins within our time series data, we have found members PPP1R12C and PPP4R2 of the protein phosphatase catalytic subunit, suggesting that HIV affects the family of phosphatases to a larger extent. With role in cytoskeleton assembly, PPP1R12C was found upregulated at protein level and downphosphorylated, while PPP4R2, involved in DNA repair, was found unchanged at protein level and downphosphorylated, with marginal downregulation at RNA level. It has been previously shown that knocking down PPP1R12C constrains early viral replication steps^2^.

The transcription factors TCF3 and TFAP4 were found differentially phosphorylated in our data. Both factors were phosphorylated and downregulated at the protein level. This behavior can either suggest that phosphorylation might be required for degradation of the protein, or that phosphorylation independently affects the activity of the protein. Downregulation and degradation of TCF3 reiterate the afore-observed inhibition at the protein level of the Wnt/β-catenin pathway during HIV replication through downregulation of proteins TCF7 and LEF1. While the phosphorylation of TCF7 and LEF1 could not be quantified due to our limit of detection, phosphorylation of TCF3 may suggest that HIV potentially inhibits the Wnt/β-catenin pathway by inducing phosphorylation of its members. The transcription factor AP4 (TFAP4), known to inhibit HIV transcription^46^, was found downregulated at the protein level in our data as well as by a previous proteomic analysis^19^. However, our data shows for the first time that, while TFAP4 is downregulated at the protein level during HIV infection, the phosphorylation status of the protein is upregulated.

The ensemble of 157 identified differentially phosphorylated proteins in our study can be retrieved and visualized on the PEACHi2 platform and constitutes a useful resource for analyzing the regulation of post-transcriptional modifications during HIV infection. However, it remains to be determined how HIV triggers up- or downphosphorylation of these proteins, as well as the roles of these phosphorylated proteins within the viral replication cycle.

## Discussion

Most studies investigating on a large scale how HIV influences the host cell during infection considered so far the cellular transcriptome at a single time point, with little attention to protein and phosphoprotein changes. Although very useful, these analyses do not capture virus-specific post-transcriptional cellular regulation and cannot identify the influence of the virus on the host cells at the protein level^47^. Recent development and increasing resolution of proteomic technologies have led to new insights in characterizing virus-host interactions at the proteome and phosphoproteome level^48^. In the present work, we employed both transcriptome and proteome high-throughput screening and reported host and virus transcriptomic, proteomic, and phosphoproteomic abundances in Mock- and HIVeGFP-infected T cells at consecutive time points of the viral replication cycle. Successive additional measurements of several viral intermediates tracing the HIV replication cycle advancement correlated with viral transcriptome and proteome dynamic quantification and delineated the time course and the main stages of the replication cycle. On the cellular host side, by clustering time series expression profiles at the three levels, we were able to identify host genes involved in HIV replication, based on their displayed protein upregulation or downregulation expression patterns. The identified temporal patterns have been compiled into an interactive resource, PEACHi2, describing the proteo-transcriptomic responses of a host immune cell to HIV infection.

Integrating proteo-transcriptomic gene expression dynamic profiles revealed 388 host genes with putative roles in HIV replication, corresponding to 192 upregulated host proteins and 196 downregulated host proteins. Over 33.5% of the identified factors have been previously reported, in support of our findings. Of note, gene expression changes induced by HIVeGFP could be attributed either to HIV or to GFP expression, as it was not possible to dissociate GFP impact in the current experimental setting. While our study recapitulated well-known virus-host interaction factors, such as CD4, CDK9, NFKB1, or UNG, it also revealed numerous novel factors which have not been described before. These new candidate factors are involved in a wide range of molecular processes, such as signaling, immune response, cell cycle, gene expression, or metabolism. The majority of these factors were not found differentially expressed at the RNA level. This may indicate, on one hand, direct induction or inhibition by HIV at the protein level, arguing for a multi-omics approach. On the other hand, it is possible that low changes in RNA expression for the majority of these genes, which were not considered significant by our analysis, may trigger stronger response at the protein level. Furthermore, noise levels, technical biases across omics technologies, as well as limit of detection at the protein level, may contribute to this effect. In contrast, HIV-mediated modulation of genes at all the three omics levels - such as for the four genes identified here, LIMA1, HIST1H1B, TFAP4, and UNG - may suggest a critical role of these genes in the HIV life-cycle. A quick, strong, and long-term response needed for these particular genes throughout the viral life-cycle could justify the need of such a multi-targeted strategy.

SILAC proteomic measurements presented inferior detection compared to transcriptome quantification. Only 26.5% of the genes detected at the RNA level were also measured successfully at the protein level, and only 4% were quantified at all three omics levels. Furthermore, the phosphoproteomic measurements contained a large amount of missing values and noise, and consequently could not be used for identifying proteo-transcriptomic expression signatures of infection. Furthermore, because phosphoprotein measurements needed to be adjusted by their protein expression in order to remove an existing abundance bias in SILAC data^49^, a large number of phosphoproteins could not be assessed due to lack of quantification at the protein level. Alternatives to SILAC, such as tandem mass tagging (TMT), could be attractive as they may yield a better coverage and especially less missing data in time series^19^. Also, combining quantifications from different proteomic techniques may increase the number of genes detected at the proteome and phosphoproteome level, and lead to more rigorous quantifications.

Time series expression profiling of the host cell during HIV-1 infection allows describing how the host gene expression is modulated through time in response to viral infection and replication. Specific temporal transcriptomic expression patterns associated to different stages of the replication cycle have been previously described with high-resolution time series analyses^18^. Our data consists of short time series (5 sampled time points per gene), which allows detection of global up- and downregulation patterns in response to infection. However, due to the relatively small number of time points and increased variability, our data is not suited for a more fine-grained stratification of expression patterns, capable for example of distinguishing early from late differential regulation. A superior number of equally distant time points, such as for example the design used in^18^, would be necessary to capture these changes robustly, as they would be supported by more than one consecutive time point.

HIV can impact the host cell at multiple levels. To alter the expression of genes at different omics levels, the virus disposes of a multitude of strategies. For example, it can affect transcription rates of genes, target RNA or protein degradation directly, or modulate enzyme activity involved in post-transcriptional and post-translational regulation. Through a multi-omics time series analysis of the host T cell gene expression, our work broadens and refines the landscape of HIV-host interactions. The different temporal expression patterns of the genes may reflect the diverse strategies of how HIV modulates host cell content. While offering a more detailed view of the host response to HIV infection, the presented analysis constitutes an initial step towards understanding the corresponding regulatory mechanisms. The identified host candidates require further validations and more targeted functional analyses in order to understand their precise role and interactions with HIV. PEACHi2 offers a reliable resource for investigating and selecting candidates for follow-up analyses. Finally, studying how interfering with these interactions affects HIV replication success may provide new insights for developing novel treatment strategies.

## Methods

### Viral infection of T-cells and sample collection

A population of 500 × 10^6^ SupT1 cells (lymphoblastic T cell line) was cultured in RPMI 1640 medium with 10% (v/v) heat-inactivated fetal bovine serum (FBS) (Invitrogen) (Fig. 1). Isotope-labeled amino acids (^13^C_6_-L-lysine, ^13^C_6_^15^N_4_-L-arginine, Cambridge Isotope Laboratories (CIL), Andover, MA) were included in the heavy (H) SILAC medium at 100 mg/l, while normal Arginine and Lysine were used in the light (L) SILAC medium. Heavy or light SILAC labeling was achieved by culturing the cells in the two media (H and L) for a minimum of 2 weeks to allow for at least 5 cell divisions. H-labeled cells were Mock infected, while the L-labeled cells were infected with an HIVeGFP/VSV-G virus at 3 μg/10^6^ cells. Infection (both Mock and with an HIVeGFP vector) was carried out by spinoculation for 30 min at 1500 g in presence of 5 μg/ml polybrene. As previously described^18^, this allowed reaching a quasi-universal infection. Cells were then washed and further incubated. The HIVeGFP viral vector used expresses GFP instead of the viral protein env. At multiple time points post-infection, cells were collected and processed for analysis of HIV life cycle progression and normalized by the 24 h time point as in^18^, as well as for transcriptome, proteome and phosphoproteome as detailed below (Supplementary File S10).

### RNA-Seq

Total RNA extraction was performed at 6 h, 12 h, 18 h, 24 h, and 30 h after Mock and HIVeGFP infection. The Illumina TruSeq protocol was used for preparing the mRNA-Seq library and cluster generation (100 NT, single end), followed by high-throughput sequencing performed on the Illumina HiSeq2000 Sequencer at the Genomics Technology Facility of the University of Lausanne. A replicate experiment was performed for the 24 h time point for both HIV and Mock conditions.

### SILAC Mass Spectrometry Protein and Phosphoprotein experiments

At 2 h, 6 h, 12 h, 18 h and 24 h post Mock and HIV infection, cells were lysed by pulse sonication in 8 M Urea, 50 mM Tris pH 7.5 and Phos-stop^TM^ phosphatase inhibitors (Roche). Clarified heavy and light extracts were quantitated and mixed 1:1. Proteins were reduced by incubation with 5 mM DTT and alkylated with 20 mM iodoacetamide, then precipitated with trichloroacetic acid/deoxycholate. Pellets were resuspended in 8 M Urea, 50 mM Ammonium bicarbonate by sonication and digested with sequencing grade trypsin (1:50) overnight at 37 **°**C. Digests were desalted on Sep-Pak C18 cartridges, and lyophilized. For total proteome analysis, digests were separated into 12 subfractions by peptide isoelectric focusing as previously described^50^. For phosphopeptide enrichment (*i.e.* peptide with phosphorylation site as a surrogate for phosphorylation status), aliquots of 1.0 mg of unfractionated digests were dissolved in loading buffer (80% acetonitrile, 5% TFA, 1 M glycolic acid) and incubated with 6 mg of titanium dioxide beads (5 μm, GL Sciences) for 10 min. The resin was washed 3x with 500 μl loading buffer, 2x with 80% acetonitrile, 0.1% TFA. A second elution was performed and analyzed separately. Phosphopeptides were eluted with 100 μl of 1% ammonium hydroxide and the eluate was immediately acidified with 1% TFA. Peptides were desalted on POROS Oligo R3 beads, dried and analyzed by LC-MS/MS. Dried peptides were resuspended in 0.1% formic acid, 2% (v/v) acetonitrile. Samples were analyzed on a hybrid linear trap LTQ-Orbitrap Velos Pro mass spectrometer (Thermo Fisher, Bremen, Germany) interfaced via a nanospray source to a Dionex RSLC 3000 nanoHPLC system (Dionex, Sunnyvale, CA, USA). Peptides were separated on a reversed-phase Acclaim Pepmap nanocolumn (75 μm ID × 25 cm, 2.0 μm, 100 Å, Dionex) with a gradient from 5 to 45% acetonitrile in 0.1% formic acid in 120 min). Full MS survey scans were performed at 60,000 resolution. All survey scans were internally calibrated using the 445.1200 background ion mass. In data-dependent acquisition controlled by Xcalibur 2.1 software (Thermo Fisher), the twenty most intense multiply charged precursor ions detected in the full MS survey scan were selected for Collision-Induced Dissociation (CID) fragmentation in the LTQ linear trap with an isolation window of 3.0 m/z in multi-stage activation mode (exciting the precursor and the neutral loss simultaneously) and then dynamically excluded from further selection during 120 s.

### Bioinformatics data analysis and statistical modeling of time series omics data

#### RNA-Seq data processing

Illumina adapter sequences were removed from RNA-Seq reads using Cutadapt v1.7.1^51^. Reads were then filtered using Prinseq-lite^52^ which removed their poly-A and low-quality (quality score less than 6) boundaries and kept for further analysis only the reads longer than 30 nucleotides and with a mean quality score larger than 20. The FastQC software^53^ was used for quality assessment of the datasets. Sequencing reads were aligned using STAR v2.5^54^ to the GRCh38 Human Genome Assembly concatenated to the HIVeGFP/VSV-G HIV genome sequence (Supplementary File S11). A standard way to construct the concatenated human/HIV genome is to add HIV as an extra chromosome to the human genome. This allows for a correct and unbiased mapping of the reads^18^. The number of reads aligned to each gene was retrieved at each time point with HTSeq-count v0.5.3^55^ with option –inverse, using the Ensembl genome annotations for human and HIV^56^ (Supplementary File S12). The read counts per gene were normalized using the median-of-ratios method introduced with the DESeq tool^57^. Genes with average read count less than 100 reads in both conditions (HIV-infected and Mock-infected) were filtered out and the gene read counts were smoothened by adding a pseudo-count of 10 reads. Afterwards, log_2_(HIV/Mock) fold changes were computed for each gene at each time point (Supplementary File S1).

Normalized relative production of total HIV reads was obtained at each time point by calculating the proportion of the HIV reads out of the total number of aligned reads at each corresponding time point (Supplementary File S10). Relative production was then obtained by adjusting the fractions to the 24 h time point.

Principal component analysis of the gene expression read counts in all the RNA-Seq samples was used to initially explore the gene expression profiles for HIV-infected and Mock-treated samples over time.

#### Mass Spectrometry SILAC data processing

Mass spectrometry data from protein and phosphoprotein experiments were analyzed and quantified using MaxQuant v1.3.0.5 (2013 release). The human subset of the release 2013_07 of the UNIPROTkb database was used (www.uniprot.org). A separate database was constructed, containing all sequences of HIV proteins and used in parallel. Cleavage specificity was Trypsin (cleavage after K, R) with two missed cleavages. Initial mass tolerances were of 4.5 particles per million (ppm) (after recalibration) for the precursor and 0.5 Da for CID tandem mass spectra. Protein and phosphopeptide identifications were filtered at 1% false discovery rate (FDR) established by MaxQuant against a reversed sequence database. Common contaminants and hits against reverse sequences were filtered out. Light/Heavy (L/H) ratios were normalized and the log_2_(HIV/Mock) fold changes at all time points were calculated. Only proteins with measurements supported by more than 2 peptides at a minimum of two consecutive time points were considered for further analysis. As suggested in^49^, the phosphoprotein ratios were normalized by their corresponding protein ratios in order to remove the bias in the phosphoprotein measurements introduced by the protein relative abundance. Therefore, only phosphoproteins with available protein measurements were considered for the analysis. Principal component analysis was used to explore the profiles of log_2_(HIV/Mock) ratios at protein and phosphoprotein levels over time.

Normalized relative production of total HIV protein expression was calculated using the intensity-based absolute quantification (IBAQ)^58^ retrieved from the SILAC MS experiment for the viral proteins. At each time point, total HIV protein IBAQ expression was divided by the total IBAQ expression from all the proteins, in order to obtain the fraction of the proteome constituted by the HIV proteins (S10 File). The fractions at each time point were further adjusted by the last time point, resulting in relative production of total HIV protein over time.

#### Differential expression analysis

An approach based on the distribution of relative expression changes between conditions(HIV-infected and Mock) as described in^59^ was employed for differential expression analysis. Accordingly, a z-score threshold of 2 standard deviations was applied to the distribution of gene log_2_(HIV/Mock) fold changes at each level (RNA, protein, and phosphoprotein) and time point. A gene was considered differentially expressed between Mock and HIV-infected conditions if, at least at one time point, its log_2_(HIV/Mock) fold change was at least two standard deviations beyond the mean of the fold changes at the corresponding time point, *i.e.*, had an absolute z-score greater than 2 at least at one time point (Supplementary Fig. S5).

#### Gaussian mixed-effects model for identifying time series gene expression patterns

Based on their log_2_(HIV/Mock) time series profiles, differentially expressed genes were clustered using a Gaussian mixed-effects model as introduced in^60^ (see the Supplementary Information for a detailed description of the model). The clustering model was implemented in the R/Bioconductor package TMixClust and is available at https://bioconductor.org/packages/release/bioc/html/TMixClust.html. Of note, we could not assess the variability of individual time point measurements due to the limited number of replicates. However, since measurements at consecutive time points are considered to be correlated, an increased number of time points is usually preferred over the availability of replicates^61^. Our statistical model is designed to capture smoothness of expression between time points and time series with highly variable behavior between consecutive time points would yield an unstable clustering result and would consequently be discarded.

We repeated the clustering procedure 50 times in order to avoid local optima and to identify the clustering configuration with the highest likelihood. We employed the distribution of the Rand index^62^ for the 50 separate clustering runs, quantifying the agreement between the different clustering solutions and the solution with the highest likelihood, in order to assess the stability of the clustering. After stability analysis for different numbers of clusters *K* = 1, …, 5, the distribution of the silhouette coefficient (or silhouette width)^63^ for each number of clusters was used to select the number of clusters that best fits the data. More precisely, the number of clusters that resulted in a clustering configuration with the largest average silhouette width was chosen.

Clustering analysis with TMixClust was applied to the time series observations of the differentially expressed genes at each data level (RNA, protein and phosphoprotein) separately. We characterized the overall temporal gene expression pattern of each resulting cluster per data type by comparing, for the ensemble of genes in each cluster, the distribution of their HIV/Mock expression throughout time to the baseline. An upregulated behavior was defined if the average temporal expression profile of the genes in a cluster was increasing and above the baseline, while a downregulation behavior corresponded to an average temporal expression profile of the genes in a cluster which was decreasing and below the baseline (Supplementary Fig. S10). The results of the clustering analysis were organized and made available through the PEACHi v2.0 R/Shiny resource available at https://peachi2.shinyapps.io/peachi2/.

#### Enrichment analysis

Enrichment analysis was performed using the Reactome pathways^21^, downloaded from MSigDB^64^, and a set of manually curated HIV-specific gene sets published in^18^. For each gene set, enrichment was tested through a hypergeometric test with Benjamini-Hochberg correction for multiple testing and a significance threshold for the corrected *p*-values of 0.05. The background gene list for the enrichment tests consisted of the ensemble of 47,339 overall expressed genes at least at one level (RNA, protein or phosphoprotein). The hierarchical structure of the Reactome pathways^21^ was used to assign enriched gene sets to major functional categories and to construct an enrichment summary.

## Electronic supplementary material

Supplementary Information

S1

S2

S3

S4

S5

S6

S7

S8

S9

S10

S11

S12

## Data Availability

The raw RNA-Seq time series data have been deposited in NCBI’s Gene Expression Omnibus^65^ and are accessible through GEO Series accession number GSE100587 (https://www.ncbi.nlm.nih.gov/geo/query/acc.cgi?acc=GSE100587). The raw proteomic and phosphoproteomic data have been deposited on the ProteomeXchange database^66^ on the PRIDE repository^67^ and are accessible through the accession number PXD005810. The source code and user manual for the Bioconductor package TMixClust is available at https://bioconductor.org/packages/release/bioc/html/TMixClust.html. The source code and data of the PEACHi2 R/Shiny resource is available on GitHub at https://github.com/cbg-ethz/PEACHi2.
