# Supplementary material for: Proteo-Transcriptomic Dynamics of Cellular Response to HIV-1 Infection
Source: Sci Rep. 2019 Jan 18;9:213. doi: 10.1038/s41598-018-36135-3 (PMC6338737; doi:10.1038/s41598-018-36135-3)
Supplement: Supplementary file 1 — Supplementary Information [file 41598_2018_36135_MOESM1_ESM.pdf]

Proteo-Transcriptomic Dynamics of Cellular Response to HIV-1 Infection

Supplementary Text, Figures, and Tables

Monica Golumbeanu [1,2], Sébastien Desfarges [3,4], Céline Hernandez [5,6], Manfredo Quadroni [5], Sylvie Rato [3], Pejman Mohammadi [7], Amalio Telenti [7]*, Niko Beerenwinkel [1,2]*, Angela Ciuffi [3]*

[1] Department of Biosystems Science and Engineering, ETH Zurich, Basel, Switzerland
[2] SIB Swiss Institute of Bioinformatics, Basel, Switzerland
[3] Institute of Microbiology, Lausanne University Hospital and University of Lausanne, Lausanne, Switzerland
[4] InvivoGen, Toulouse, France
[5] Center for Integrative Genomics, University of Lausanne, Lausanne, Switzerland
[6] Computational Systems Biology Team, Institut de Biologie de l'Ecole Normale Supérieure, CNRS UMR8197, INSERM U1024, ENS, PSL Université, Paris, France
[7] Department of Integrative Structural and Computational Biology, The Scripps Research Institute, La Jolla (CA), USA
* corresponding authors

Supplementary Text:

Time series clustering using a Gaussian mixed-effects model

The ensemble of observed $\log_2$(HIV/Mock) expression ratios $X_{i,j}$, for each gene i at each time-point j, constitutes a time series $\boldsymbol{X_i} = (X_{i,1}, X_{i,2}, ..., X_{i,T})$, where T is the total number of time points. TMixClust separates the time series into K groups, based on their dynamic patterns. To do so, the random variables $X_{i,j}$ of each time series $\boldsymbol{X_i}$ belonging to cluster k are modeled as linear combinations of a mean expression baseline at time point j, $\xi_k(j)$, describing the general dynamic pattern within cluster k (fixed effect), a gene-specific effect β_i invariant over time, accounting for a shift of the expression of gene i from the baseline (random effect), and an error term $\varepsilon_{i,j}$,

$$(\boldsymbol{X_{i,j}} \mid \boldsymbol{k}) = \boldsymbol{\xi}_k(\boldsymbol{j}) + \boldsymbol{\beta}_i + \boldsymbol{\varepsilon}_{i,j}$$

The gene effect β_i and error $\varepsilon_{i,j}$ are modeled with Gaussian distributions: $\beta_i \sim N(0, \theta_k)$ and $\varepsilon_{i,j} \sim N(0, \delta)$, where $N(\mu, \sigma)$ denotes the Gaussian distribution with mean μ and standard deviation σ. This parametrization results in a corresponding multivariate Gaussian model for each time series observation belonging to cluster k: $P(\boldsymbol{X_i}|k) \sim N(\boldsymbol{\xi_k}, \Sigma_k)$, with $\boldsymbol{\xi_k} = (\xi_k(1), \xi_k(2), ..., \xi_k(T))$. The elements of the covariance matrix Σ_k are expressed as functions of θ_k and δ as described in [1] and in the TMixClust package vignette.

To set up the clustering problem, we model the time series $\boldsymbol{X_i}$ as a mixture of multivariate Gaussian distributions with mixing coefficients π_k,

$$\boldsymbol{X_i} \sim \sum_{k=1}^{K} \pi_k N(\boldsymbol{\xi_k}, \Sigma_k)$$

and use an expectation-maximization algorithm to estimate maximum likelihood model parameters. Instead of choosing a parametric form for the baseline $\boldsymbol{\xi_k}$, we adopt a less restrictive, nonparametric approach using smoothing splines. Gu *et al.* have shown that when fitting smoothing splines to a set of Gaussian random variables, the residual sum of squares (RSS) minimization problem can be

naturally transformed into a maximum-likelihood formulation [2]. Once the model parameters are estimated, the probability that gene i belongs to cluster k is used to assign genes to clusters. Specifically, gene i is assigned to the cluster k with

$$k = \text{argmax}_k P(\text{gene } i \in \text{cluster } k) = \text{argmax}_k \frac{\pi_k N(\boldsymbol{X_i} \mid \boldsymbol{\xi_k}, \Sigma_k)}{\sum_{k=1}^{K} \pi_k N(\boldsymbol{X_i} \mid \boldsymbol{\xi_k}, \Sigma_k)}$$

where $N(\boldsymbol{X_i} \mid \xi_k, \Sigma_k)$ represents the multivariate Gaussian density function of $\boldsymbol{X_i}$ with mean $\boldsymbol{\xi_k}$ and covariance matrix Σ_k.

Supplementary Figures:

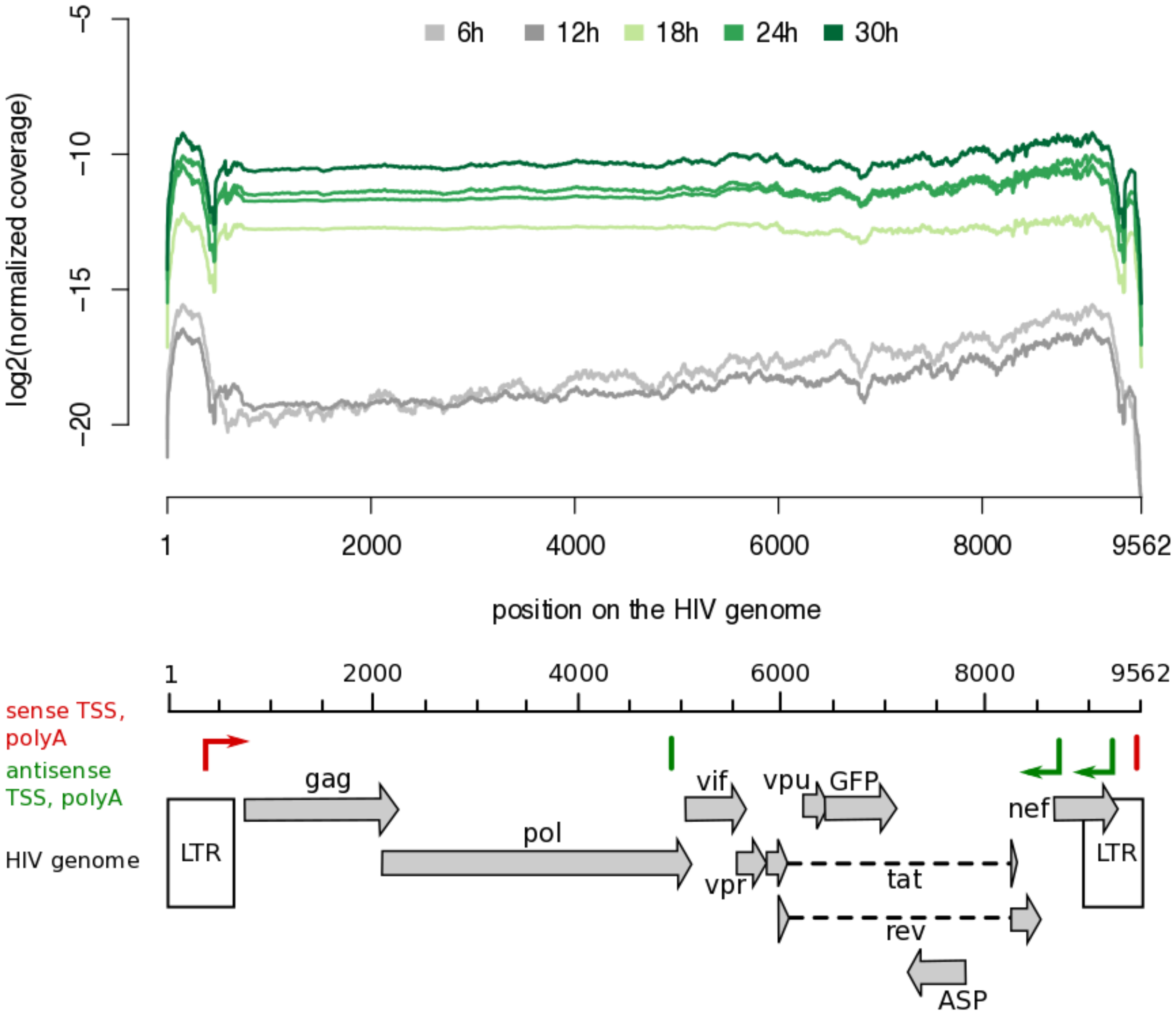

Figure S1. HIV read coverage analysis following RNA-Seq experiments in HIV-infected SupT1 cells. To calculate the normalized coverage, the number of sequencing reads aligning at each position on the HIV genome was adjusted by library size and log2-transformed. The corresponding HIV gene annotations and their genomic positions are indicated below the coverage plot. A significant increase in HIV RNA material at 18h was observed, consistent with the late phase of HIV replication.

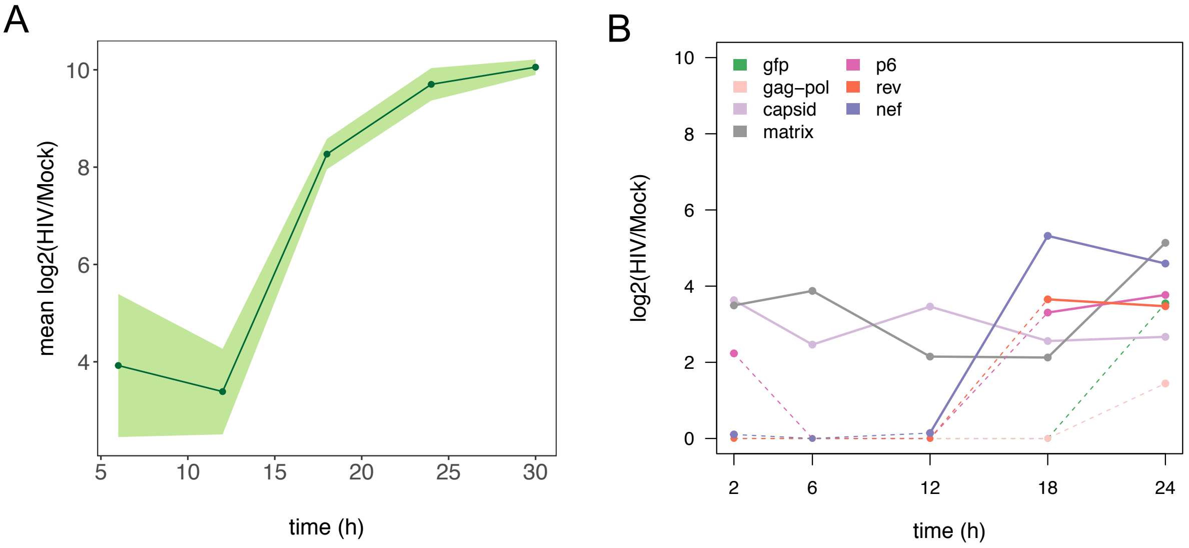

Figure S2. Time series analysis of HIV transcriptome and proteome. (A) Considering the log2(HIV/Mock) ratios of all the viral transcripts, we computed the total average viral transcript expression over time (dark green points and line) and corresponding variability (one standard deviation, light green shade). The slight decrease in viral transcriptome material between 6h and 12h can be explained by existence of viral RNA from the initial infection at 6h. The late phase in HIV replication is marked with a significant increase of the average viral transcript expression at 18h. **(B)** HIV/Mock protein ratios were obtained by detection of HIV-specific peptides by mass spectrometry (MS) and compared between HIV and background (Mock-infected cells). The presence of p6, matrix and capsid proteins at early time points can be explained by their abundance from the initial infection input which persists in the infected cells and is more difficult to distinguish from *de novo* viral production. For the other viral proteins, such as nef and rev, low levels from the initial infection inoculums are detectable, and *de novo* production was detected at the 18h and 24h time points, consistent with viral synthesis occurring during the late phase of the replication cycle.

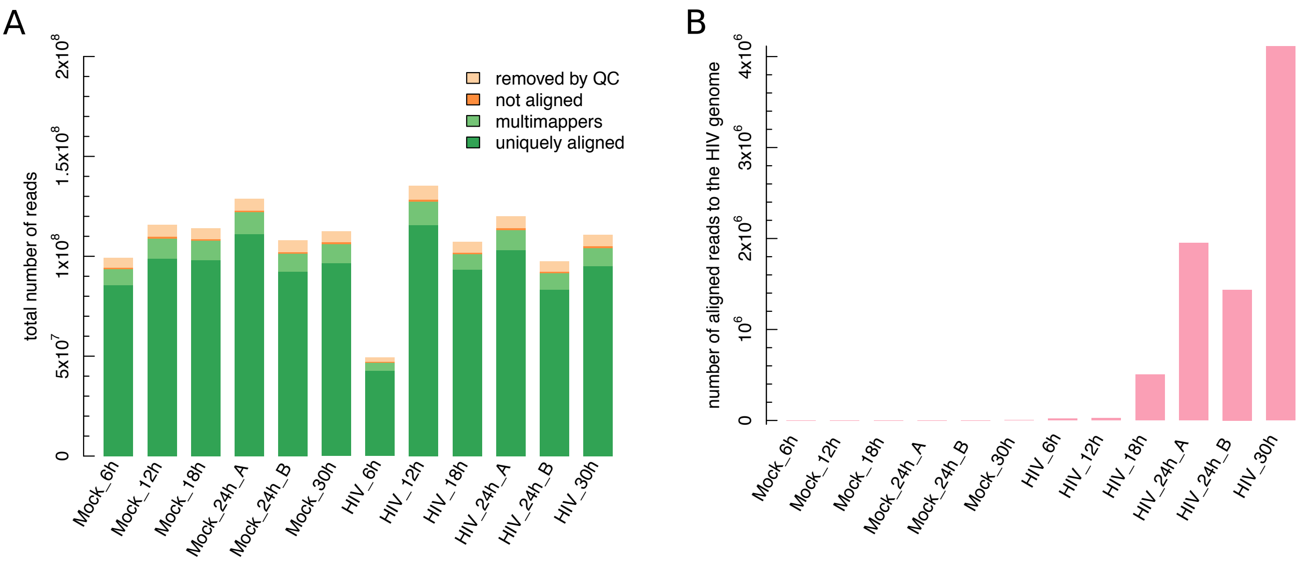

Figure S3. Overview of RNA-Seq read pre-processing and alignment results. (A) Quantitative analysis of RNA-Seq read library sequence processing and alignment, showing the proportions of reads removed by quality control (QC) as well as the proportions of uniquely aligned and multiply-aligned reads. **(B)** Number of sequencing reads aligned to the HIV genome at each time point in Mock and HIV-infected cells.

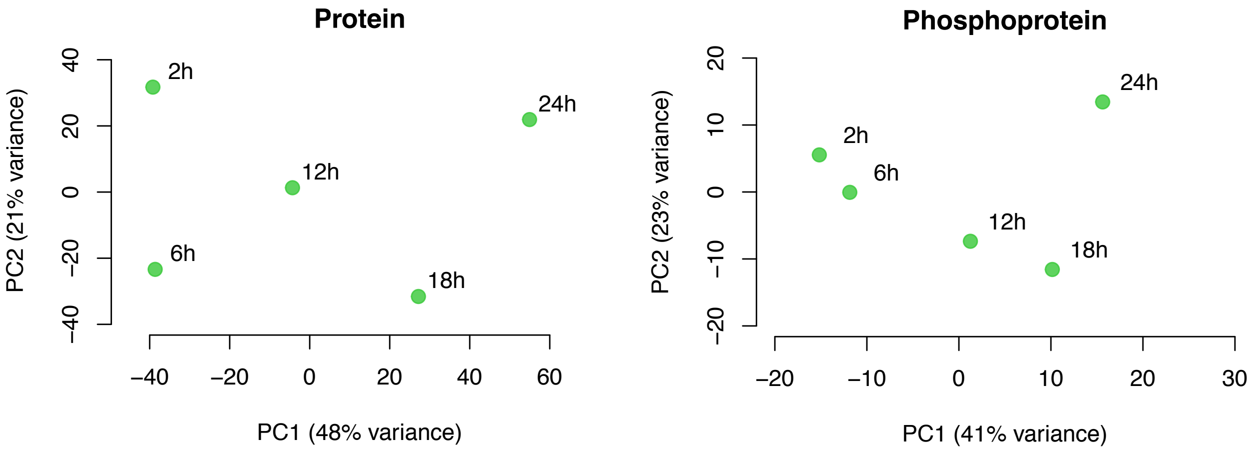

Figure S4. Principal component analysis of the log2(HIV/Mock) protein and phosphoprotein expression profiles over time. Each data point corresponds to the set of protein (left) and phosphoprotein (right) log2(HIV/Mock) ratios at each corresponding time point indicated by the attached label. In each case, the first principal component (PC1, x axis) explains variance in a time-dependent manner. The percentages of explained variance are indicated within the labels of the axes.

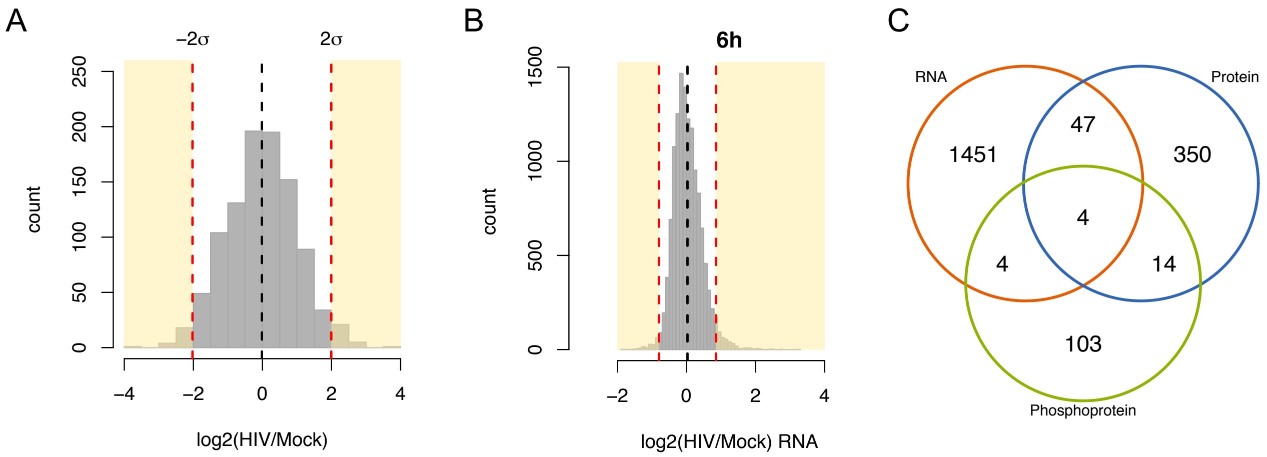

Figure S5. Differential expression analysis of time-series omics data. (A) Symbolic representation of the z-score-based approach for differential expression analysis. The grey bars represent the distribution of log2(HIV/Mock) fold changes for a particular data type (RNA, protein or phosphoprotein) and time point, while the black dotted line marks the mean of the distribution. To determine differentially expressed genes, a threshold of 2 standard deviations (red lines) was set. Therefore, all genes whose log2(HIV/Mock) ratios have an absolute z-score larger than 2 (yellow regions) were considered differentially expressed at the corresponding time point. **(B)** Example of differential expression analysis on the RNA-Seq data at 6h. The distribution of log2(HIV/Mock) fold changes of RNA measurements at 6h is represented by the grey bars. Its mean (black dotted line) and 2 standard deviation thresholds (z-score=2, red dotted lines) are specified. The genes whose log2(HIV/Mock) ratios at 6h were located in the yellow regions were considered differentially expressed at 6h. A gene is globally considered differentially expressed if it is found differentially expressed at least at one time point. **(C)** Venn diagram presenting the numbers of detected differentially expressed genes and their overlap at RNA, protein, and phosphoprotein levels.

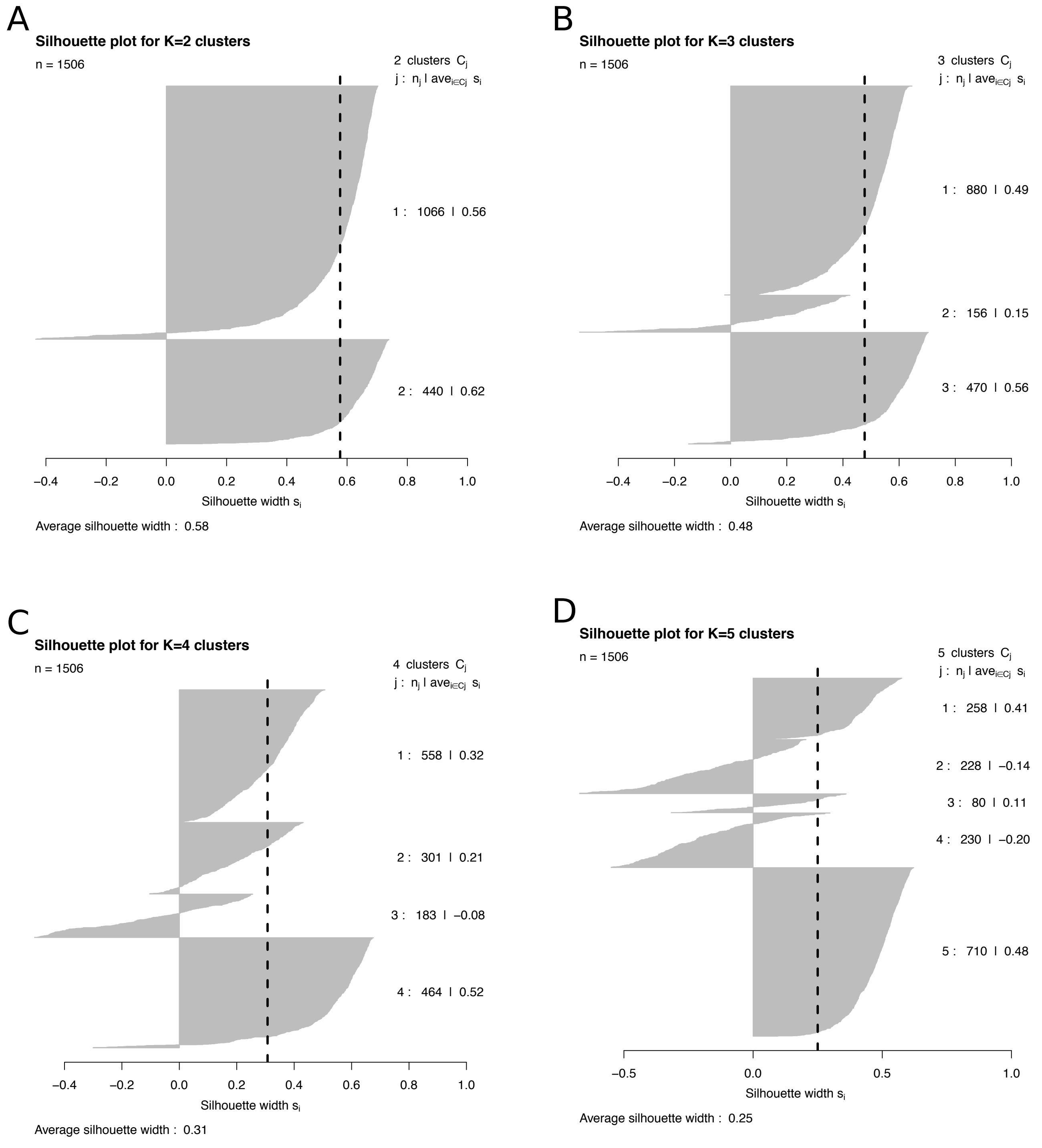

Figure S6. Silhouette analysis for time series RNA-Seq data clustering. To determine the optimal number of clusters that best stratifies the time series patterns in RNA-Seq data, the distribution of the silhouette coefficients (silhouette widths, grey bars) was investigated for different clustering configurations: K=2 **(A)**, K=3 **(B)**, K=4 **(C)**, and K=5 **(D)** clusters. Only data points with complete observations were considered for calculating the silhouette coefficients. The largest average silhouette width (dotted line) was obtained with K=2 clusters and started to decrease with increasing K. Each silhouette plot also specifies on the right-hand side the number of genes in each cluster, as well as the average silhouette width in the corresponding cluster. The global average silhouette width is specified on the lower left corner of each plot.

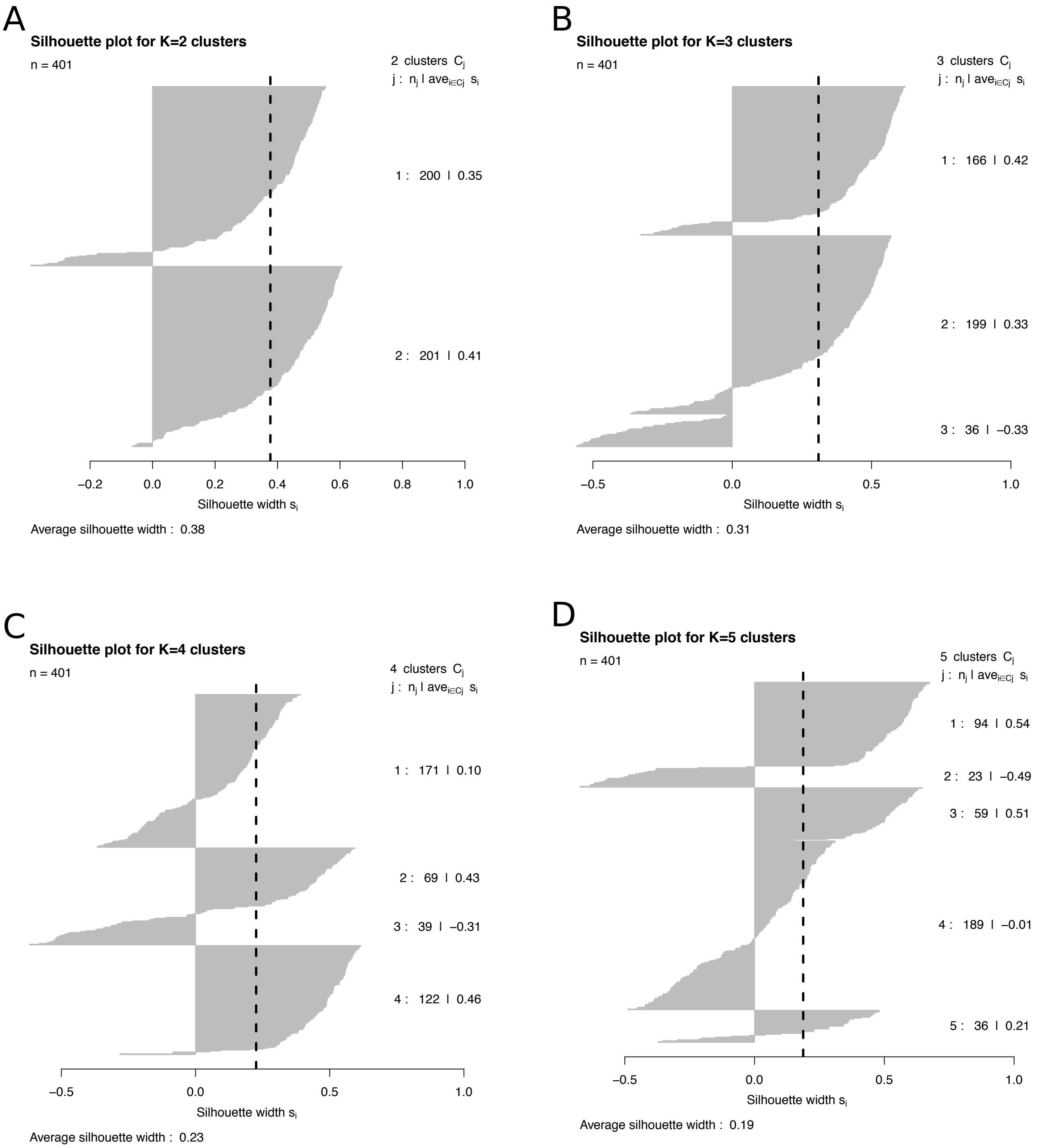

Figure S7. Silhouette analysis for time series proteomic data clustering. To determine the optimal number of clusters that best stratifies the time series patterns in the proteomic data, the distribution of the silhouette coefficients (silhouette widths, grey bars) was investigated, for different clustering configurations: K=2 **(A)**, K=3 **(B)**, K=4 **(C)**, and K=5 **(D)** clusters. Only data points with complete observations were considered for calculating the silhouette coefficients. The largest average silhouette width (dotted line) was obtained with K=2 clusters and started to decrease with increasing K. Each silhouette plot also specifies on the right-hand side the number of genes in each cluster, as well as the average silhouette width in the corresponding cluster. The global average silhouette width is specified on the lower left corner of each plot.

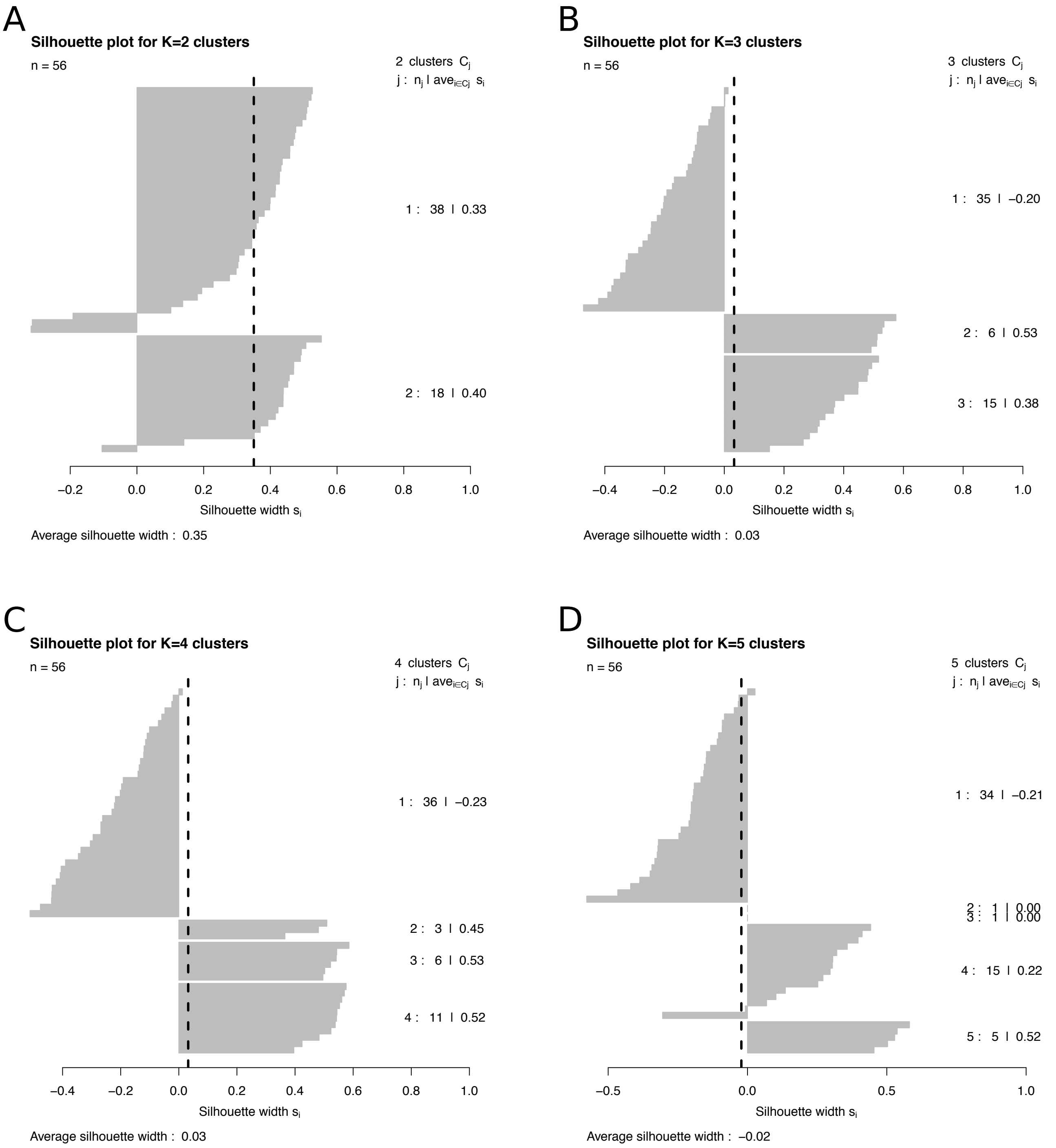

Figure S8. Silhouette analysis for time series phosphoproteomic data clustering. To determine the optimal number of clusters that best stratifies the time series patterns in phosphoproteomic data, the distribution of the silhouette coefficients (silhouette widths, grey bars) was investigated, for different clustering configurations: K=2 **(A)**, K=3 **(B)**, K=4 **(C)**, and K=5 **(D)** clusters. Only data points with complete observations were considered for calculating the silhouette coefficients. The largest average silhouette width (dotted line) was obtained with K=2 clusters and started to decrease with increasing K. Each silhouette plot also specifies on the right-hand side the number of genes in each cluster, as well as the average silhouette width in the corresponding cluster. The global average silhouette width is specified on the lower left corner of each plot.

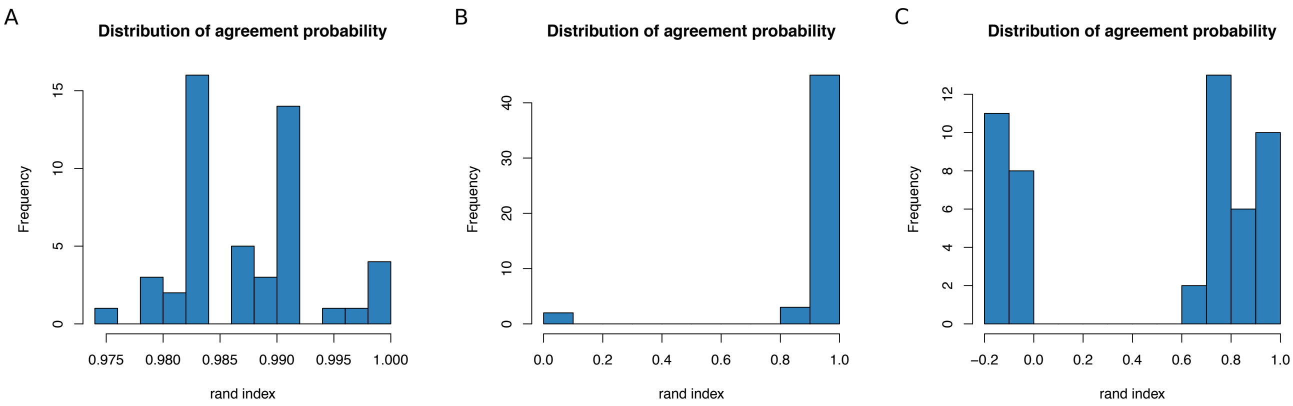

Figure S9. Stability analysis of time series omics data clustering for K=2 clusters. The distribution of the rand index is informative for the stability of the inferred optimal solution of the EM clustering scheme. Precisely, it shows how often the algorithm reaches the solution with the maximum likelihood across several clustering runs with the same number of clusters K (in this case, 50 clustering runs with K=2). For the RNA-Seq data **(A)**, all runs yielded solutions which were more than 97% identical to the solution with the highest likelihood across all the clustering runs. The protein clustering runs **(B)** also reported in more than 90% of the cases the overall maximum likelihood solution. Clustering of the phosphoprotein data **(C)** proved to be less stable, due to the large amount of noise and missing values in the data, only 10 out of 50 runs reached the solution with the highest likelihood.

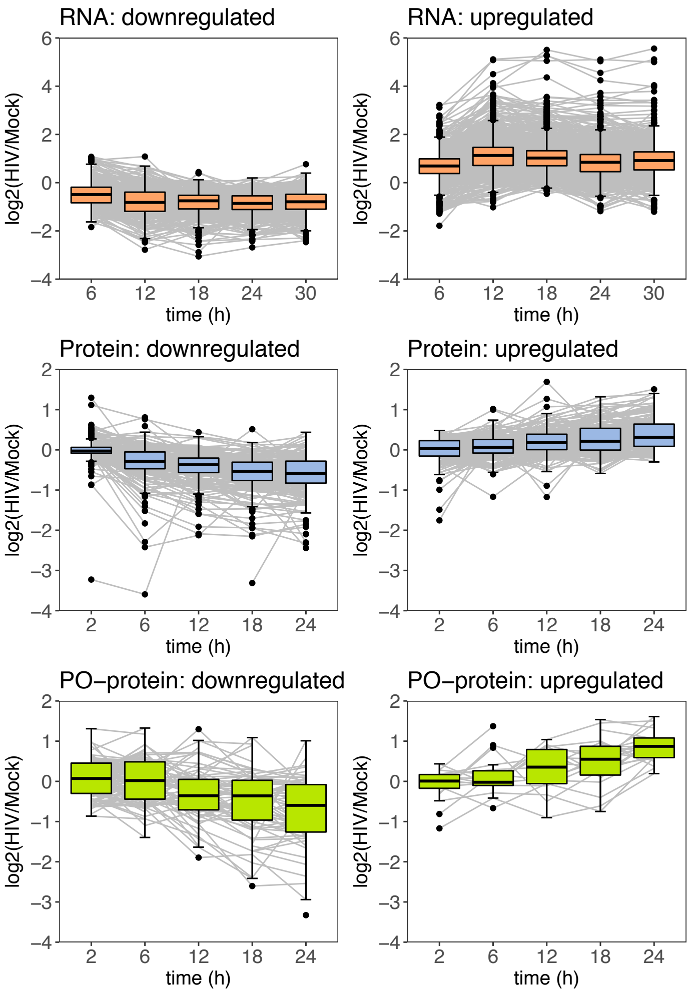

Figure S10. Clustering of time series omics data. The clustering procedure implemented in the R/Bioconductor package TMixClust was applied to RNA **(upper panels)**, protein **(middle panels)** and

phosphoprotein **(lower panels)** time series data. Stability and silhouette analysis showed that a number of K=2 clusters corresponded to the best stratification for all the three data types, separating main upregulation and downregulation patterns. Each figure depicts the time series expression profiles associated to each cluster. Accordingly, every gene expression time series is represented by a grey curve and, for every time point, an associated box plot summarizes the expression distribution for RNA (orange), protein (blue) and phosphoprotein (green) in each cluster. PO-protein: phosphoprotein.

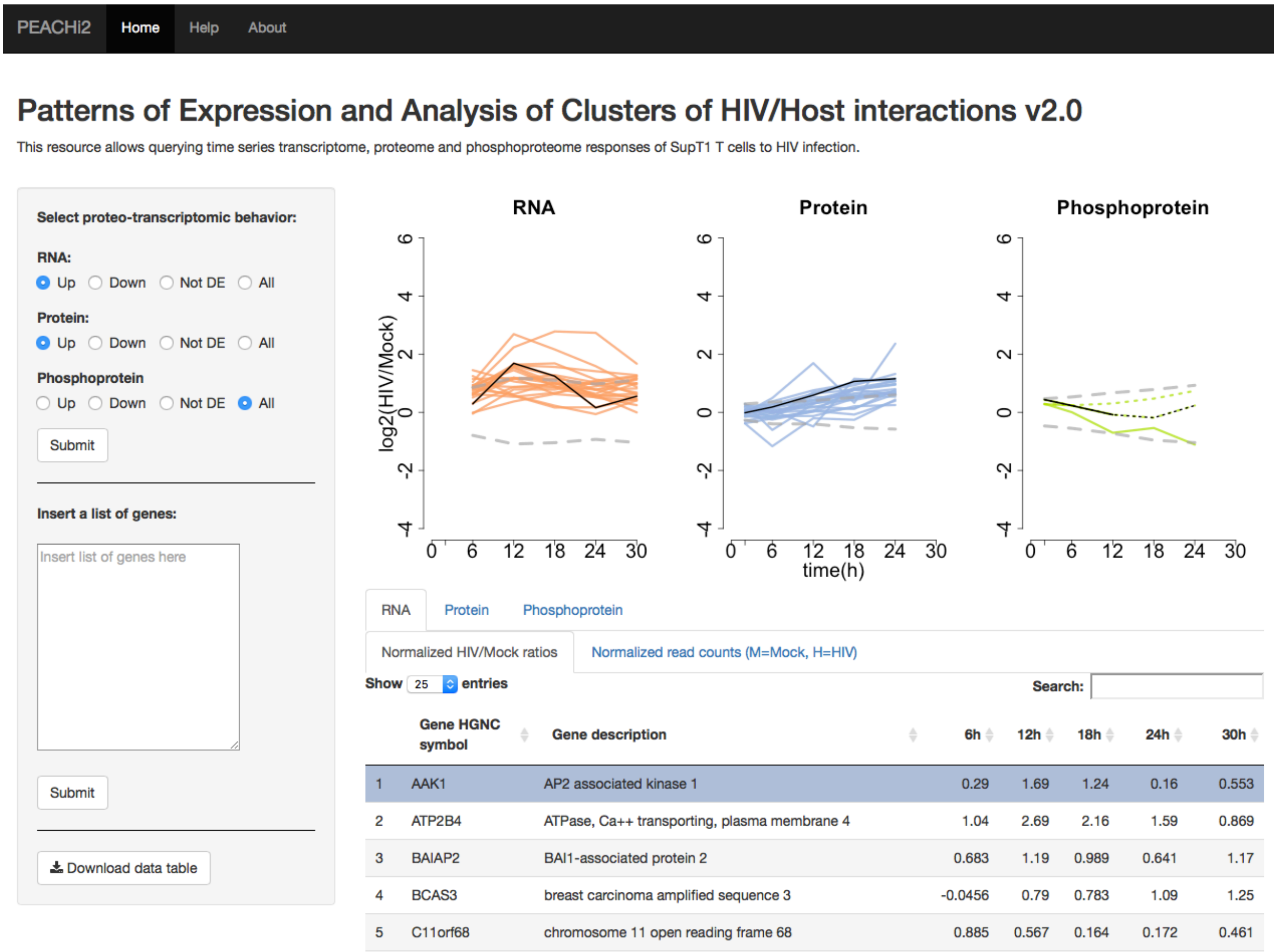

Figure S11. PEACHi2 platform for querying time series proteo-transcriptomic behavior of host genes to HIV-1 infection. Main page of the R/Shiny application. On the left panel, the user can either select the desired behavior at RNA, protein and phosphoprotein levels, or specify a list of genes in the empty text area. “Up” stands for upregulated, “Down” stands for downregulated, “Not DE” stands for not differentially expressed and “All” corresponds to any behavior. The right panel displays the results of the user query, namely, the time series plots at each data level, as well as the corresponding data tables containing the gene names, gene description and data measurements. The selected gene in the data table is highlighted on the time series plot.

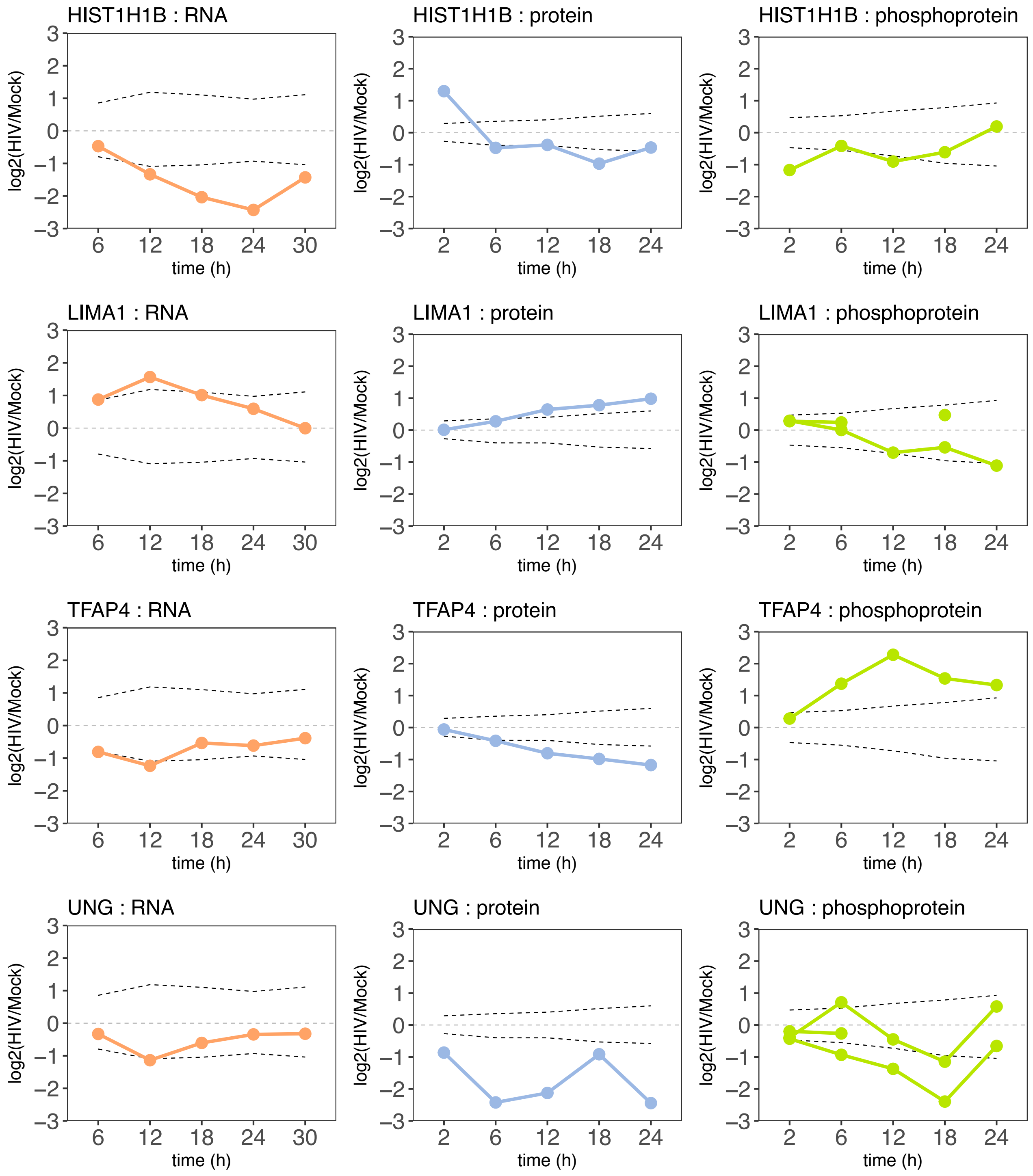

Figure S12: Genes differentially expressed at all the three omics levels. Four genes presented differential expression behavior at RNA, protein and phosphoprotein level, namely HIST1H1B, LIMA1, TFAP4, and UNG. Their corresponding time series behaviors are represented at each level (colored curves), as well as the level-specific fold change cutoffs used for differential expression (black dotted line). The grey dotted lines mark the zero fold changes.

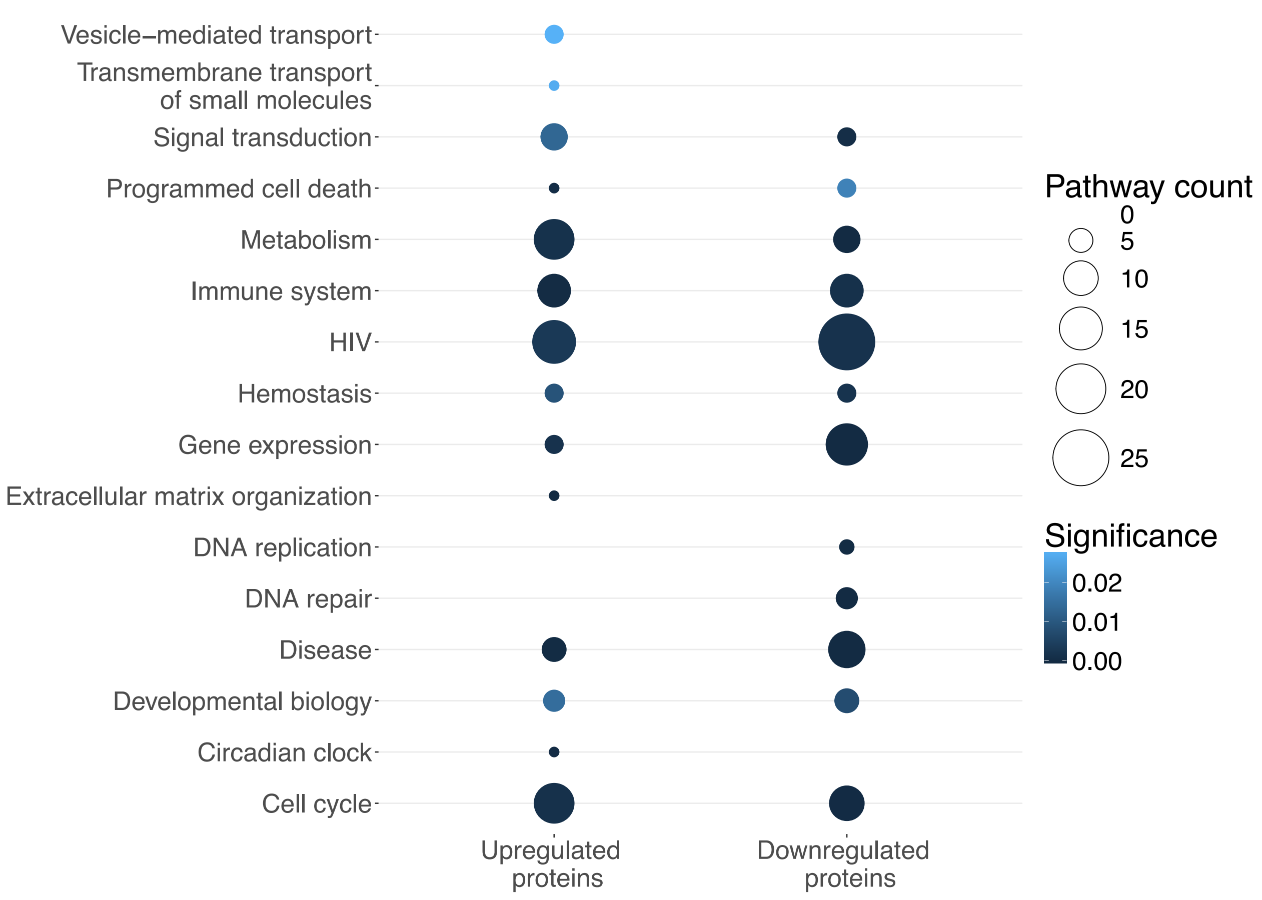

Figure S13. Enrichment analysis of putative host factors involved in HIV replication. The isolated putative factors were analyzed in terms of enrichment in Reactome pathways, as well as in HIV-related pathways included in a separate collection (S4 File). Enriched Reactome pathways were grouped according to the Reactome hierarchy into enriched categories, while the HIV-related pathways were assigned to a separate category called HIV. Category names are specified on the left side of the figure. Each circle size (Pathway count) is proportional to the number of enriched pathways included in the corresponding category. The color of each circle (Significance) reflects the geometric mean of the corrected *p*-values for the pathways included in each category.

Supplementary Tables:

Table S1. Quantitative results of RNA-Seq data preprocessing and alignment.

Sample name	Uniquely aligned	Multimappers	Not aligned	Removed by QC
Mock 6h	85,571,802	8,069,531	712,713	4,937,322
Mock 12h	98,816,156	10,138,863	770,614	5,948,125
Mock 18h	98,091,746	9,728,254	790,683	5,493,378
Mock 24h rep. 1	111,074,327	10,952,284	903,123	5,962,354
Mock 24h rep. 2	92,268,569	9,031,651	797,857	5,922,226
Mock 30h	96,670,207	9,577,571	783,081	5,445,970
HIV 6h	42,792,263	4,078,610	333,448	2,229,765
HIV 12h	115,744,958	11,551,674	977,667	6,915,267
HIV 18h	93,321,788	7,802,764	785,648	5,408,141
HIV 24h rep. 1	103,087,557	10,076,683	848,273	5,938,392
HIV 24h rep. 2	83,175,452	8,414,852	765,091	5,204,788
HIV 30h	94,960,156	9,258,641	777,130	5,732,870
**Average**	**92,964,581**	**9,056,781**	**770,444**	**5,428,216**

The table contains the number of RNA-Seq reads obtained after each step in the data processing pipeline and alignment to the human genome. Each column following the sample name corresponds to a step of the processing pipeline. The last row specifies the rounded average of each corresponding column.

Table S2: Quantitative results of omics profiling experiments.

	RNA level	Protein level	Phosphopeptide level
**Initially detected (Raw data)**	31,660 genes (13 viral genes)	4,947 proteins (7 viral genes)	2,072 phosphopeptides (793 unique proteins)
**Host genes selected after filtering and normalization**	13,057 genes	3,613 proteins	1,111 phosphopeptides (590 unique proteins)
**Host differentially expressed genes (z = 2)**	1,506 genes (11.53%)	415 proteins (11.48%)	157 phosphopeptides (125 unique genes) (14.13%)

For each data level (RNA, protein or phosphoprotein), the table displays the number of initially detected genes, as well as the number of remaining genes after filtering steps and the number of differentially expressed genes. The percentages correspond to the proportion of differentially-expressed genes out of the total number of filtered genes.

Table S3: Previously-published studies proposing candidate host genes involved in HIV-1 replication.

Publication	Description	Reported genes	Upregulated host factors	Downregulated host factors	Total intersection
Brass et al. 2008	Genome-wide functional siRNA screen	281	3	8	11
Cleret-Buhot et al. 2015	Microarray transcriptional profiling	1778	18	23	41
Jager et al. 2012	Protein-protein interaction assay	522	14	14	28
Konig et al. 2008	Genome-wide siRNA screen	295	6	6	12
McLaren et al. 2015	*In silico* analysis of evolutionary signatures associated with HIV restriction	56	2	1	3
Navare et al. 2012	Proteomic screen	141	4	14	18
Schoggins et al. 2011	Overexpression screening	331	4	1	5
Wojcechowskyj et al. 2013	Phosphoproteomic screen	175	4	5	9
Yeung et al. 2009	siRNA screen	262	3	3	6
Zhou et al. 2008	siRNA screen	291	4	5	9
Zhu et al. 2014	siRNA screen	84	1	2	3
Mohammadi et al. 2013	Curated list of genes involved in HIV-related molecular processes	2194	38	36	74
	**Total of unique genes:**	**3743**	**57**	**73**	**130**

Each row of the table corresponds to a published study presenting putative host genes interacting with HIV during the viral replication cycle. For each study, we report the intersection between the corresponding list of genes and our identified upregulated and downregulated host factors.

References

1. Ma P, Castillo-Davis CI, Zhong W, Liu JS. A data-driven clustering method for time course gene expression data. Nucleic Acids Research. 2006;34(4):1261-9. doi: 10.1093/nar/gkl013. PubMed PMID: PMC1388097.

2. Gu C. Smoothing Spline ANOVA Models: R Package gss. Journal of Statistical Software; Vol 1, Issue 5 (2014). 2014. doi: 10.18637/jss.v058.i05.
